# Chromic Acid

**Published:** 1869-07

**Authors:** 


*Chromic Acid.*—Dr. E. Magitot in *Bulletin General de la Therapeutique*, advocates the use of *chromic acid* in various affections of the mucous membrane of the mouth, such as general and ulcerative stomatitis, gingivitis, aphthae, &c, For alveolo-dental periostitis this writer specially recommends it as having a rapid and beneficial effect.

Chromic acid is prepared as follows: To 100 parts, by measure of cold saturated solution of bichromate of potassa, 150 parts of pure sulphuric acid are added and allowed to remain till cool; the sulphuric acid unites with the potassa, and the chromic acid crystallizes in deep red needles—very soluble and deliquescent.

It possesses powerful oxidizing and bleaching properties, and acts as a solvent of organic matter, in view of which it is necessary to use it with caution, Its principal use in medicine is a caustic application care being taken that it does not spread beyond prescribed limits, as there is no danger of its doing when properly managed. When used as a caustic, as soon as its corrosive action is finished, it passes into the state of inert pulverent sesquioxide.

When applied to mucous membrane it should be diluted with two or more parts of water; in this proportion it has been successful in arresting uterine hœmorrhage.

